# Effect of Aging Treatment on the Strength and Microstructure of 7075-Based Alloys Containing 2% Li and/or 0.12% Sc

**DOI:** 10.3390/ma16237375

**Published:** 2023-11-27

**Authors:** Ali Tahmasbi, Agnes M. Samuel, Yasser Zedan, Victor Songmene, Fawzy H. Samuel

**Affiliations:** Department of Mechanical Engineering, École de Technologie Supérieure, Montreal, QC H3C 1K3, Canada; ali.tahmasbi6574@gmail.com (A.T.); agnesmsamuel@gmail.com (A.M.S.); yasser.zedan.1@gmail.com (Y.Z.); victor.songmene@etsmtl.ca (V.S.)

**Keywords:** 7075 alloys, Li addition, Sc addition, aging treatment, microhardness, phase precipitation

## Abstract

The present study was performed on three versions of 7075 alloy to which Sc or Sc + Li was added. The alloys were subjected to various aging treatments. The microhardness results show that the highest value of hardness was achieved when the alloy containing Li + Sc was aged at 120 °C for 24 h whereas the minimum level was exhibited by the base alloy aged at 280 °C. The results were interpreted in terms of the size and distribution of the main hardening phase (η′(MgZn_2_)), and the role of the presence of Al and Cu in the used alloy. Precipitation of Al_3_(Sc, Zr, Ti) phase particles during solidification of the Sc-containing ingots was also discussed. The coarsening and spheroidi-zation of η-phase particles take place through the Ostwald ripening mechanism while smaller par-ticles in solution dissolve and deposit on larger particles. In Sc-containing alloys, star phase particle consists of different layers. The change in the brightness from layer to layer indicates that the Zr and Sc concentrations are varied within the star phase, since the atomic number of Zr (40) is higher than the atomic number of Sc (21). The addition of Sc, as well, leads to marked decrease in the grain size of the as-cast alloys i.e., 300 µm and 45 µm, respectively. The interaction between Li and Sc would reduce the effectiveness of the grain refining effect of Sc. The results of the refining effect of Sc were confirmed using the EBSD technique.

## 1. Introduction

Artificial age hardening of the 7075-type alloys is considered the main source of alloy strength. Several aging procedures were reported in the literature: single aging treatment (aging at 120 °C for certain aging times [1,2,3,4,5]) and double aging (two-step-process at two different aging temperatures [6,7]). According to the authors, increasing the concentration of the η_1_ phase (MgZn_2_) particles following double aging heat treatment was effective in improving the alloy strength. After 10 h of double aging, the alloy toughness level was increased by about 300% in comparison with the single aging treatment. Retrogression and re-aging (RRA) heat treatments composed of three steps, where step 1 and 3 are the same, also received appreciable interest. It has been suggested that this process is the most influential factor in age hardening in the sense that applying a higher retrogression temperature would lead to increasing the degree of dissolution and more stable precipitates on re-aging [8,9,10]. It is suggested that the heat treatment process follows the sequence saturated solid solution → GP zones → ή (MgZn_2_) → η (MgZn_2_). The orthorombic S (Al_2_CuMg) phase and the T (Al_32_(Mg,Zn)_49_) phase may also form [11,12,13].

Alloying elements play a significant role in contributing to the strength of Al–Zn–Mg–Cu-based alloys. It has been reported by Tai et al. [14] that 7075 alloy containing 0.24% Mn possesses high ultimate tensile strength coupled with low ductility due to enrichment of dispersoids with Mn which may enhance the transformation from GPII to η′ phase. Several authors investigated the effect of the addition of Sc and Zr to 7075 alloy. Their results showed that 7075 alloy, 7075 alloy + Zr, or 7075 alloy + Sc + Zr alloys offer better mechanical properties caused by the precipitation of Al_3_(Sc, Zr) dispersoids [15,16,17,18].

The addition of Li to 7075 alloys was also examined in other studies [19,20,21]. Following T6 treatment of Li-containing 7075 alloy, T1, S, and also fine δ′ (Al_3_Li) phase particles with a considerable amount of δ′ would result in significant improvement in the alloy strength in the peak-hardening condition [19,21]. The addition of Mg accelerates the precipitation rate in 7075 alloys. The combined introduction of Ag, Mg, and Zn was found to further enhance the precipitation rates without much change in the sequence of phase precipitation [22]. Also, adding Ag and Zn to the alloy results in a marked increase in the alloy strength. In the case of Ag-containing alloys, T and η phases were observed in the microstructure. It is suggested that the precipitation sequence can be expressed as SSS → η–type cluster → GP zone → η′ → η → T since Ag promotes the precipitation of the T-phase [23].

The objective of this research article is to investigate the influence of scandium (Sc) and lithium (Li) additions, in combination with different heat treatment conditions, on the type of phase precipitation and their role in determining the strength of the Al 7075 alloy. This has been carried out using scanning electron microscopy (SEM) and energy dispersive spectroscopy (EDS) techniques which provide valuable insights into the microstructural variations and elemental composition of the alloy subjected to these different conditions.

The second goal pursued in this study was to determine the maximum and minimum hardness values obtained from the five heat treatments used for the studied alloys. To achieve this objective, three aluminum alloys including Aluminum 7075, Aluminum 7075-Sc (with 0.1% Sc addition), and Aluminum 7075-Sc-Li (with 0.1% Sc and 2.2% Li addition) were used. All alloys were subjected to various aging treatments. The results are presented in this article.

## 2. Experimental Procedure

**Figure 1:**
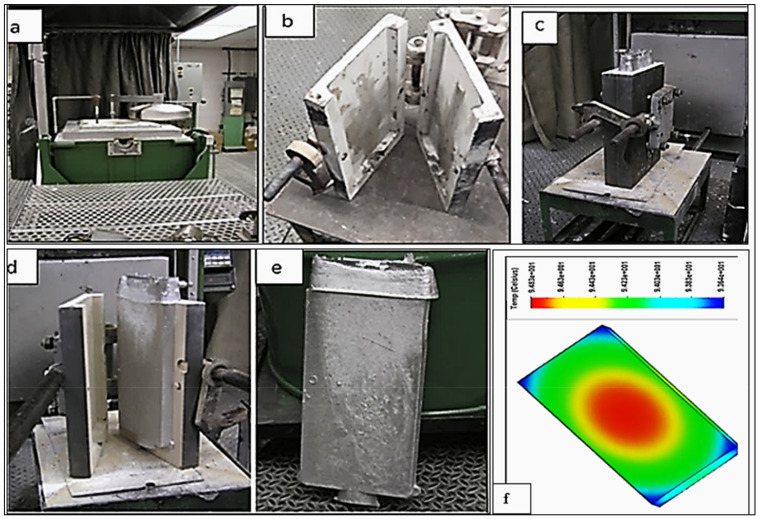
Casting of ingots of alloys used in the present study showing the (**a**) melting furnace, (**b**) metallic mold prior to casting, (**c**) metallic mold with casting, (**d**) open mold showing casting inside, (**e**) actual casting (dimensions of each block are 38 × 170 × 330 mm), and (**f**) temperature distribution. Casting weight is about 8 kg and was solidified at the rate of 0.15 °C/s.

The Al–Li alloy was received in the form of Al-4 wt.% Li master alloy. Alloying elements were added in the form of pure Cu, Al-2 wt.% Sc master alloy, pure silver (Ag), commercially pure Al (99.5%), and Al-15%Zr master alloy to molten commercial 7075 alloy to produce the composition of the Al 7075 alloy (coded A). Al-2%Sc master alloy was added to alloy A to obtain alloy Al 7075-Sc (coded B) and to alloy B to produce Al7075-Li-Sc alloy (coded C), as shown in Table 1. Prior to casting, the melting temperature was maintained at 750 ± 5 °C. In each case, the melts were degassed for ~15–20 min with a rotary graphite impeller rotating at ~130 rpm, using pure dry argon to ensure the complete removal of oxides, inclusions, and hydrogen. Following this step, the melt was carefully skimmed to remove oxide layers from the surface. Three samplings for chemical analysis were also taken simultaneously at the time of the casting; this was performed at the beginning, in the middle, and at the end of the casting process to ascertain the exact chemical composition of each alloy. These samplings were poured into 600 °C preheated graphite molds to obtain close to equilibrium solidification conditions. Before pouring, a K-type (chromel–alumel) thermocouple was inserted into the center of the mold, to record the temperature–time data during solidification, for purposes of identifying the reactions taking place during solidification and for microstructural examination [24]. Figure 1 shows details of the castings prepared from the three alloys used. Samples from these alloys were cut and heat treated using a forced-air Blue M Electric furnace equipped with a programmable temperature controller (±2 °C). All samples were solutionized at 470 °C for a period of 4 h before quenching in warm water (~60 °C). The quenching rate was about 50 °C/s.

Vickers hardness measurements were carried out using an automatic CLEMEX micro-hardness tester (Clemex, Brossard, QC, Canada) and an indentation load of 100 gf was applied for 10 s. Prior to testing, the machine was calibrated using a sample (standard) of well-known hardness. A matrix of 10 × 10 indentations was carried out on each specimen to analyze the hardness distribution. The distance between two indents was approximately 1000 µm. Scanning electron microscopic (SEM) analysis was carried out on ground and polished un-etched samples (that were mounted individually in bakelite to obtain a mirror-like surface), employing a Hitachi SU-8230 FESEM (Hitachi High Tech Corporation, Ibaraki, Japan) equipped with a Bruker Quantax Flat Quad EDS detector (Bruker AXS LLC, Madison, WI, USA) to investigate phase evolution in the heat-treated and reference samples. For a deeper interpretation of the grain structure, an a EBSD analysis was carried out on polished samples. However, within the limitation of the technique used, it may be recommended that a more sensitive XRD technique could be used to enable a more accurate analysis of the phases.

## 3. Results and Discussion

The hardness (i.e., strength) of the alloy material resulting from the heat treatment regime applied will be discussed for the three Al 7075 alloy types studied, namely alloys Al 7075, Al 7075-Sc, and Al 7075-Sc-Li. The effect of the Sc and Li alloying element additions to the base Al 7075 alloy would be automatically included together with that of the heat treatment: the additions will affect the precipitates formed during each of the heat treatments applied, which, in turn, will affect the hardness exhibited by the alloy in question. As the precipitates formed are microscopic in size, the strength/hardness is measured in terms of microhardness, using a Vickers microhardness tester.

## 4. Effect of the Heat Treatments on the Microhardness of Al 7075 Alloy

**Figure 2:**
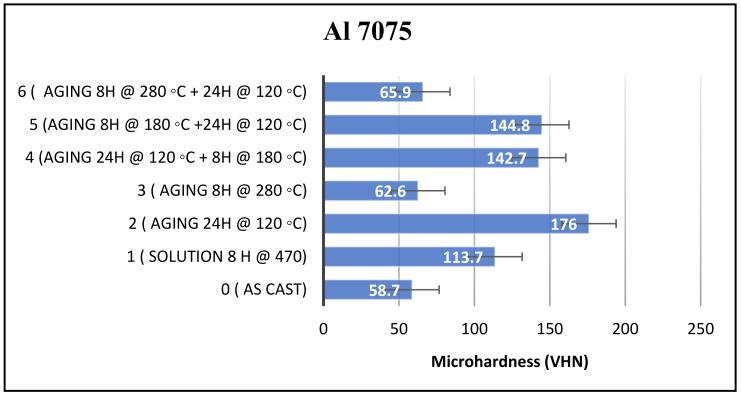
Microhardness of Al 7075 samples subjected to different heat treatments.

The measured microhardness results for Al 7075 alloy are displayed in Figure 2. Based on the findings, it was observed that subjecting the Al 7075 alloy to a heat treatment process at a temperature of 120 °C for a duration of 24 h resulted in the highest microhardness level, namely 176 VHN. In contrast, employing a heat treatment process at a temperature of 280 °C for a period of 8 h (treatment #3) resulted in the lowest microhardness level for this alloy among all the heat treatment processes used, measuring approximately 62.6 VHN.

Solution heat treatment (SHT) had a notable effect on enhancing the microhardness of the as-cast alloy. The initial microhardness of the as-cast alloy, measured at 58.7 VHN, was improved considerably, giving a microhardness of 113.7 VHN. This represents a significant improvement of 55 VHN units compared to the as-cast condition.

In order to examine the impact of double aging on microhardness, three distinct double aging processes were tried on the alloy. In one of these processes, the sample was initially aged at 280 °C for 8 h, followed by a subsequent aging at 120 °C for 24 h (treatment #6). The resulting microhardness measurement showed a value of 65.9 VHN, displaying a value comparable to that obtained through single aging at 280 °C for 8 h (treatment #3), namely, 62.6 VHN.

With respect to the other two double aging treatments, the microhardness value obtained from double aging for 24 h at 120 °C and 8 h at 180 °C (treatment #4) was very close to the microhardness value obtained when the sequence of the double aging process was reversed, viz., using 8 h at 180 °C followed by 24 h at 120 °C (treatment #5). The microhardness values in these two cases were 142.7 VHN and 144.8 VHN, respectively. Overall, from the results obtained for the three double aging treatments, it is reasonable to conclude that the aging temperature is the factor that controls the resulting microhardness rather than the aging time. In addition, aging at 120 °C is more effective in increasing the alloy strength than 180 °C. Using a temperature as high as 280 °C drastically reduces the alloy strength due to the commencement of over-aging, with either a single aging or double aging treatment. This suggests that the precipitates are affected to different extents at these different temperatures. An examination and analysis of the corresponding microstructures using SEM/EDS, as will be discussed later in the Section on SEM/EDS investigations, would shed further light on how and why the observed microhardness values result.

## 5. Effect of the Heat Treatments on Microhardness of Al 7075-Sc Alloy

**Figure 3:**
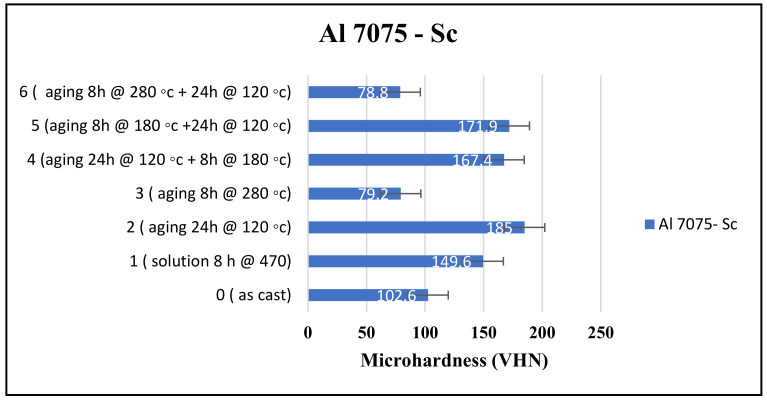
Microhardness of Al 7075-Sc samples obtained with different heat treatments.

In this study, the microhardness of the as-cast Al 7075-Sc was significantly higher than that of the Al 7075 sample—see Figure 3. The reason for this phenomenon is due to the distinct compositions of the two alloys. Table 1 illustrates that Al 7075-Sc contains 0.11% Sc in addition to 6.6% Zn, which is almost 1 percent higher than Al 7075 which has 5.62% Zn. The results of the study by Pruthvi and Shenoy [25] also indicated that the weight percentage of Zn has the most significant contribution to hardness, accounting for approximately 47.05% of the variations observed. Therefore, it is crucial to precisely control and take into consideration the amount of Zn added to achieve the desired hardness value in the material.

In another study, Li and coworkers [26] discovered that the addition of a small amount of Sc leads to the formation of Al_3_(Sc and Zr) particles, which play a crucial role in refining the cast microstructures of Al–Zn–Mg–Cu–Zr-based alloys. Their findings indicated that the most optimal and practical amount of Sc addition in such alloys is 0.21 wt.%. Therefore, by adding 0.21 wt.% Sc, they were able to achieve the desired microstructural refinement in the Al–Zn–Mg–Cu–Zr-based alloys investigated in their work. In the present study, although the microhardness was higher in the as-cast Al 7075-Sc alloy sample in comparison to the as-cast Al 7075 alloy, the Al 7075-Sc alloy reacted similarly to the different heat treatment processes in each case.

The Al 7075-Sc alloy exhibited the highest microhardness value of 185 VHN when subjected to a single aging process for 24 h at 120 °C. This aging treatment matches the widely recognized T6 temper, which is known to render 7075 aluminum alloys very hard. The T6 heat treatment involves a solution heat treatment within the temperature range of 450–480 °C followed by aging at 120–185 °C. During artificial aging, precipitates such as Al_2_CuMg and MgZn_2_ form, contributing to the increased hardness [27]. Due to Sc–Zr interaction leading to the formation of the complex compound (Al_3_(Sc, Zr, and Ti)), the effectiveness of both additives in terms of increasing the alloy hardness was compromised.

However, it is important to note that higher aging temperatures can lead to the formation of larger precipitates resulting in a decrease in alloy hardness. This observation was also evident in our study where the microhardness decreased to 79.5 VHN after aging for 8 h at 280 °C. Interestingly, despite achieving the highest microhardness through single aging at 120 °C for 24 h, it was unable to compensate for the hardness reduction. Even this condition resulted in a slightly lower microhardness of 78.8 VHN though this difference is negligible.

**Figure 4:**
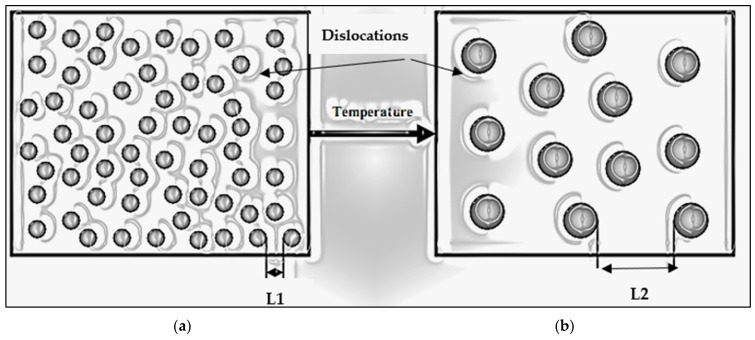
Effect of increasing the aging temperature on the size and inter-particle distances of the phase hardening precipitates: (**a**) low aging temperature and (**b**) high aging temperature. (L1 and L2 are the inter-particle distance in (**a**,**b**), respectively).

In this alloy, the microhardness resulting from the double aging treatment of 24 h at 120 °C followed by 8 h at 180 °C was equal to 167.4 VHN. The reverse process (8 h at 180° C and then 24 h at 120 °C, treatment #6) also did not show a meaningful difference since the resulting microhardness was 171.9 VHN, close to the former. This interesting observation again reinforces the fact that the temperature is the main factor controlling the hardness obtained, as discussed previously. Figure 4 schematically shows the effect of increasing the aging temperature on the size and distribution of the precipitated phase particles.

## 6. Effect of the Heat Treatments on Microhardness of Al 7075-Li-Sc Alloy

The importance that aluminum–lithium (Al–Li) alloys have gained in the aerospace industry stems from such valuable properties as their low density, high stiffness, and an excellent strength-to-weight ratio. When Li is added to high-strength alloys, it brings about a reduction in the material density and an increase in the elastic modulus. As a result, Al–Li alloys offer enhanced performance for aerospace applications where lightweight and strong materials are essential [28].

**Figure 5:**
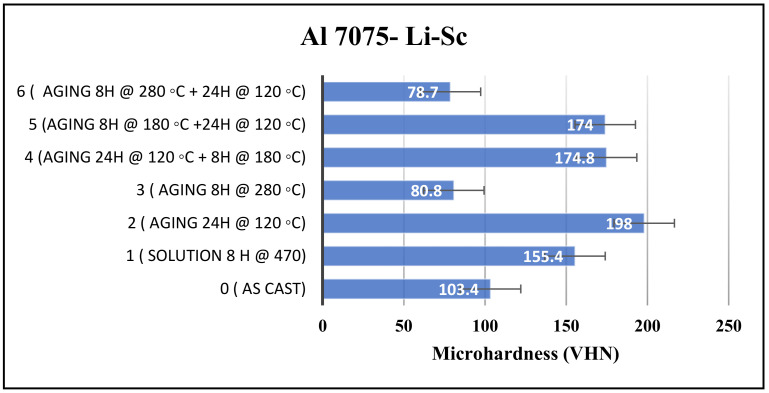
Microhardness of Al 7075-Sc-Li samples obtained with different heat treatments.

In order to investigate the influence of alloying the Al-7075 alloy with Li on the microhardness, as a mechanical property criterion, 2.2 wt.% Li was added to Al 7075-Sc alloy. The microhardness results, shown in Figure 5, revealed that this addition did not significantly affect the microhardness of the alloy in the as-cast condition. The microhardness of the Al 7075-Sc-Li alloy, measured at 103.4 VHN, was only slightly higher than that of Al 7075-Sc (102.6 VHN).

Furthermore, during the conducted heat treatment processes, both alloys exhibited almost no difference in their microhardness. For instance, at the almost lowest microhardness obtained after aging for 8 h at 280 °C, both alloys exhibited approximately 80 VHN. Double aging of 8 h at 280 °C followed by 24 h at 120 °C resulted in identical microhardness values of 78 VHN for both alloys. Similarly, aging at 180 °C for 8 h followed by 24 h at 120 °C also yielded the same results, with only a slight variation measured at 174 VHN. The reverse process produced exactly the same outcome.

From the Li-Sc binary diagram constructed based on a schematic phase diagram for Li-rare earth systems [29], 2% Li would react with Sc at a temperature as high as 1500 °C. While the addition of Li did not play a significant role in altering the microhardness under most heat treatment conditions, it became more notable when aiming for the highest microhardness achieved by aging at 120 °C for 24 h. In this condition, the microhardness reached 198 VHN, nearly 13 units higher than that of the Al 7075-Sc alloy sample alone. This finding suggests that lithium is highly effective in the formation of small and uniformly dispersed precipitates, contributing to the enhanced microhardness of the Al 7075-Sc-Li alloy.

## 7. SEM/EDS Investigations of Al 7075-Li-Sc Alloys

**Figure 6:**
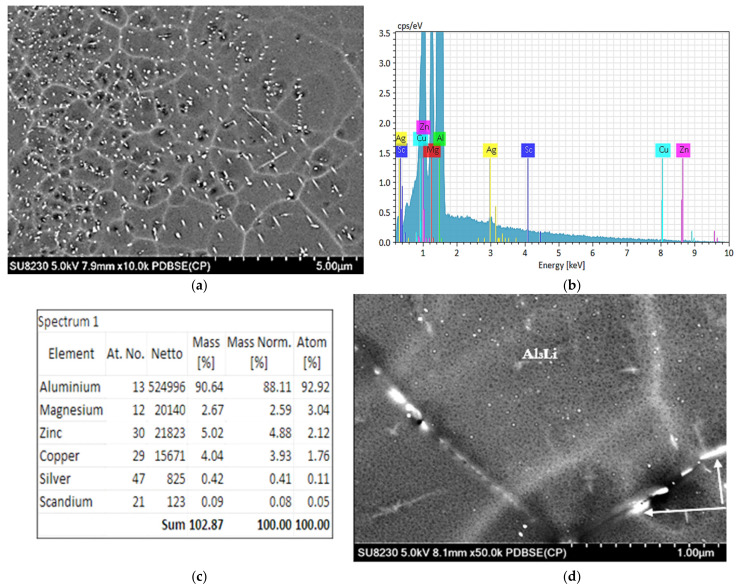
(**a**) Backscattered electron image obtained from an (Al 7075-Li-Sc) alloy sample aged at 120 °C for 24 h; (**b**,**c**) EDS spectrum and analysis, respectively, of the particles in (**a**), (**d**) precipitation of Al_3_Li phase particles not the absence of PFZ in this case coupled with precipitates of Zr-rich phase particles at the grain boundary (arrowed).

After the heat treatment process of the solid solution at 120 °C for 24 h, the precipitated phases in the alloy consisted mainly of Al_3_(Sc, Zr), η′ (MgZn_2_), and θ’(Al_2_Cu) phases. The η′ phase is the most abundant metastable phase in the artificially-aged alloy and is the primary strengthening precipitate; it appears typically as small and spherical particles. These particles are uniformly distributed throughout the aluminum matrix and can be observed in SEM images as small spots (Figure 6a). The EDS analysis provided in Figure 6b shows the average chemical analysis obtained from Figure 6a as a mixture of Al_2_Cu and MgZn_2_ phase particles whereas Figure 6c reveals the precipitation of ultrafine particles of the Al_3_Li phase.

**Figure 7:**
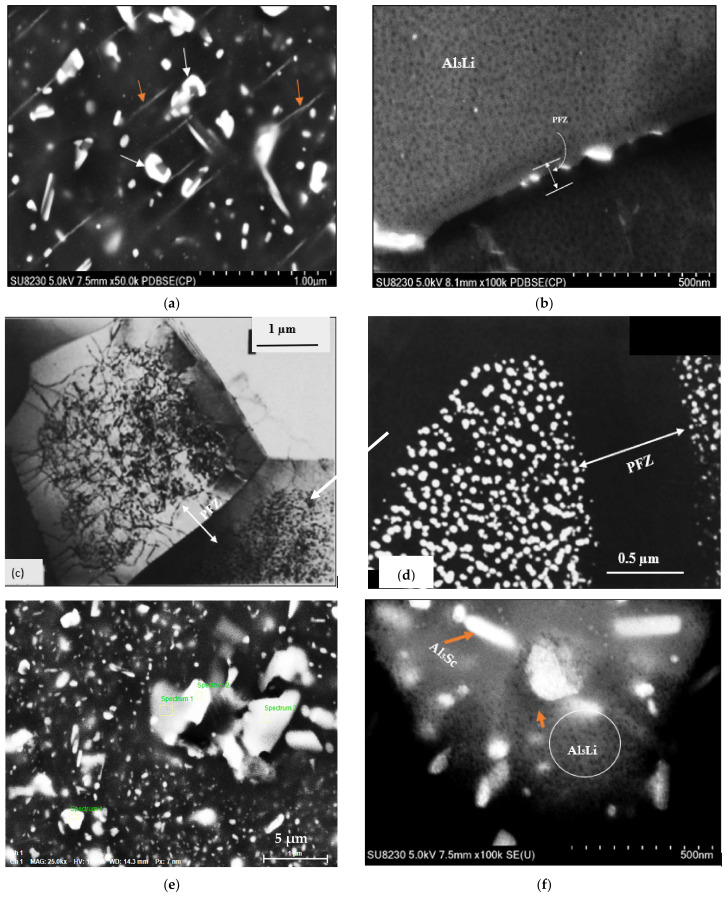

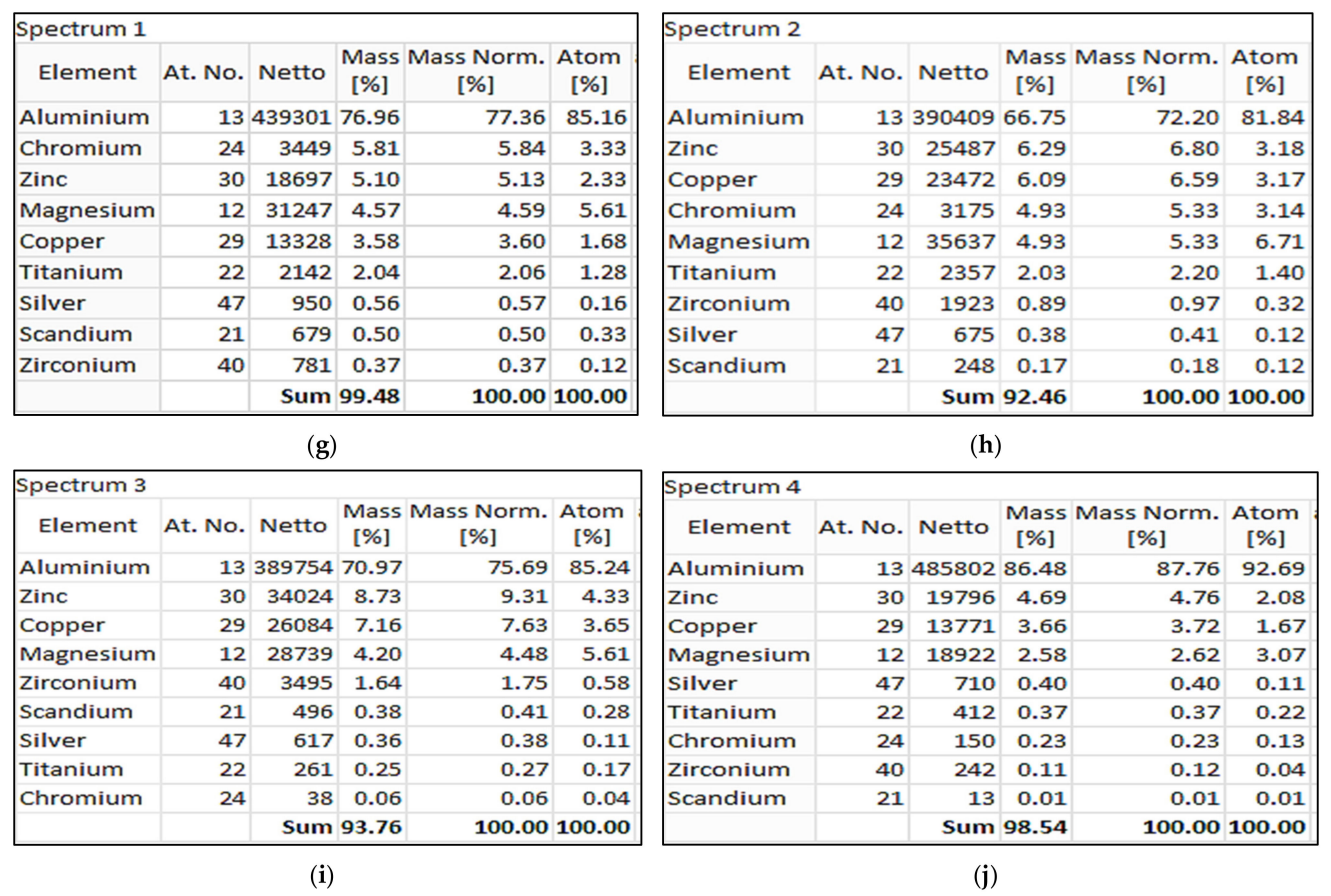
(**a**) High magnification backscattered electron image of an (Al 7075-Li-Sc) alloy sample aged at 280 °C for 8 h showing coarsening and spheroidization of the white phase particles (white arrows) at the cost of dissolution of the other particles (orange arrows); (**b**) high magnification image of (**a**) revealing the precipitation of what could be Al_3_Li phase particles; (**c**) bright field TEM micrograph of Al_3_Li (δ′) phase particles in binary Al-2.5%Li treated similarly, (**d**) dark field TEM image of (**c**), (**e**) BSE image the same as in (**a**) taken at low magnification; (**f**) backscattered electron image showing the precipitation of Al3Li (black spots) observed in the alloy in the T7-tempered condition; (**g**) EDS analysis of spot 1 in (**b**); (**h**) EDS analysis of spot 2 in (**b**); (**i**) EDS analysis of spot 3 in (**b**); (**j**) EDS analysis of spot 4 in (**b**).

Comparing Figure 6 and Figure 7 shows that the precipitates are smaller and more uniform in Figure 5, which is obviously the reason for the increase in hardness in the Al 7075-Li-Sc alloy when aged at 120 °C for 24 h. The application of heat treatment to aluminum alloys can result in the creation of distinct precipitates and phases characterized by diverse shapes and microstructures. The exact nature of the microstructure will depend on the specific heat treatment process used. The addition of alloying elements plays a vital role in enhancing the characteristics of Al–Zn–Mg–Cu alloys and the control of the types and amounts of these added elements is implemented during the evolution of these alloys [30]. The precipitates that form in 7075 alloy include the η′ (eta-prime), η (eta), and S’ (S-prime) phases (Figure 7a). The η′ phase is the primary strengthening precipitate in 7075 alloy. It forms at around 120–160 °C and reaches peak strength at around 120 °C. The η phase forms at higher temperatures of around 160–200 °C and has a lower strengthening effect than the η′ phase. The S’ phase forms at even higher temperatures of around 200–250 °C and is less common in 7075 alloy [31]. Figure 7g,h reveals the presence of two phases/particles in Figure 7e: a gray phase containing 5.13% Zn, 4.6% Mg, and 3.6% Cu and a white phase containing 6.8% Zn, 5.33% Mg, and 6.59% Cu. Due to the low atomic number of Li, it is difficult to picture it clearly, as shown in Figure 7b, compared to δ’precipitates shown in Figure 7c—bright field-and its corresponding dark field image depicted in Figure 7d produced using high resolution TEM microscopy [32,33]. 

The addition of lithium to the 7000 series aluminum alloys can significantly alter the precipitation behavior during artificial aging. The Li modifies the alloy microstructure by reducing the solubility of Cu in aluminum, promoting the formation of a new strengthening phase, called the T1 phase or theta phase. The T1 phase typically forms between 100 °C and 160 °C, while the η′ phase forms at around 120–160 °C in standard 7075 alloy. The T1 phase has a similar strengthening effect as the η′ phase but is smaller and more homogenously distributed in the matrix. The combination of T1 and η′ phases leads to a synergistic strengthening effect in these alloys (Champier and Samuel, 1986) [32]. Figure 7h reveals the precipitation of ultra-fine particles that could be of Al_3_Li phase. As the atomic number of Li is 3, it is very difficult to identify the exact nature of the phase with certainty.

The addition of both Li and Sc to 7000 series aluminum alloys can further modify the precipitation behavior during artificial aging. Scandium is known to enhance the precipitation kinetics of aluminum alloys and promote the formation of fine and homogeneously distributed precipitates. When both Li and Sc are added to the alloy, it can exhibit even higher strength and toughness than with Li alone. The addition of Sc promotes the formation of a new intermediate precipitate, called the β″ (beta double prime) phase, which acts as a nucleation site for the T1 and η′ phases. The β″ phase forms at lower temperatures than the T1 phase, typically between 60–100 °C [34].

**Figure 8:**
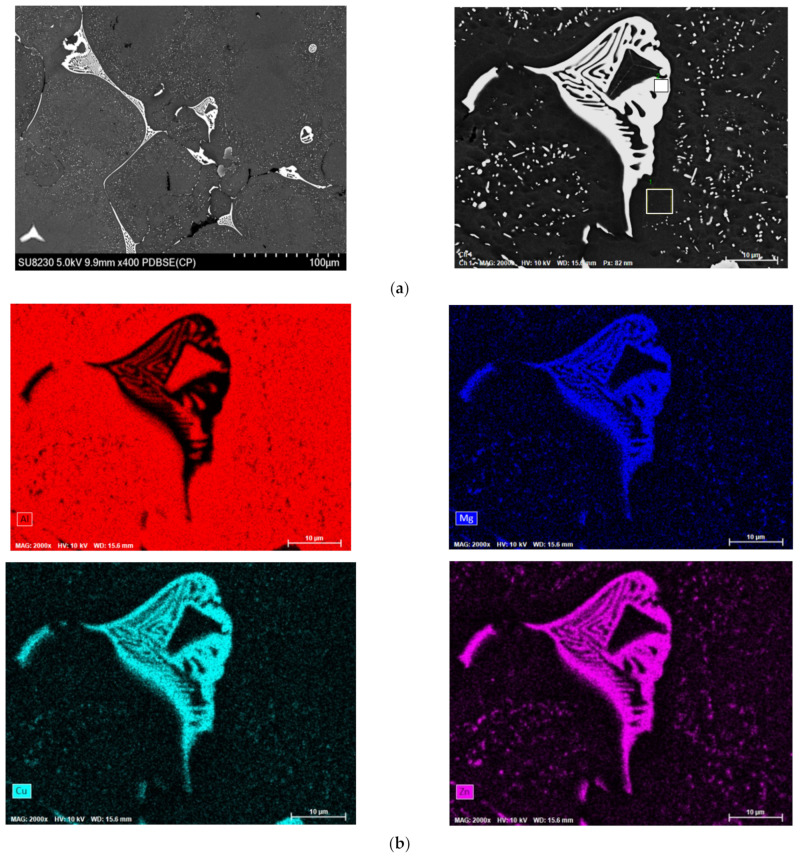

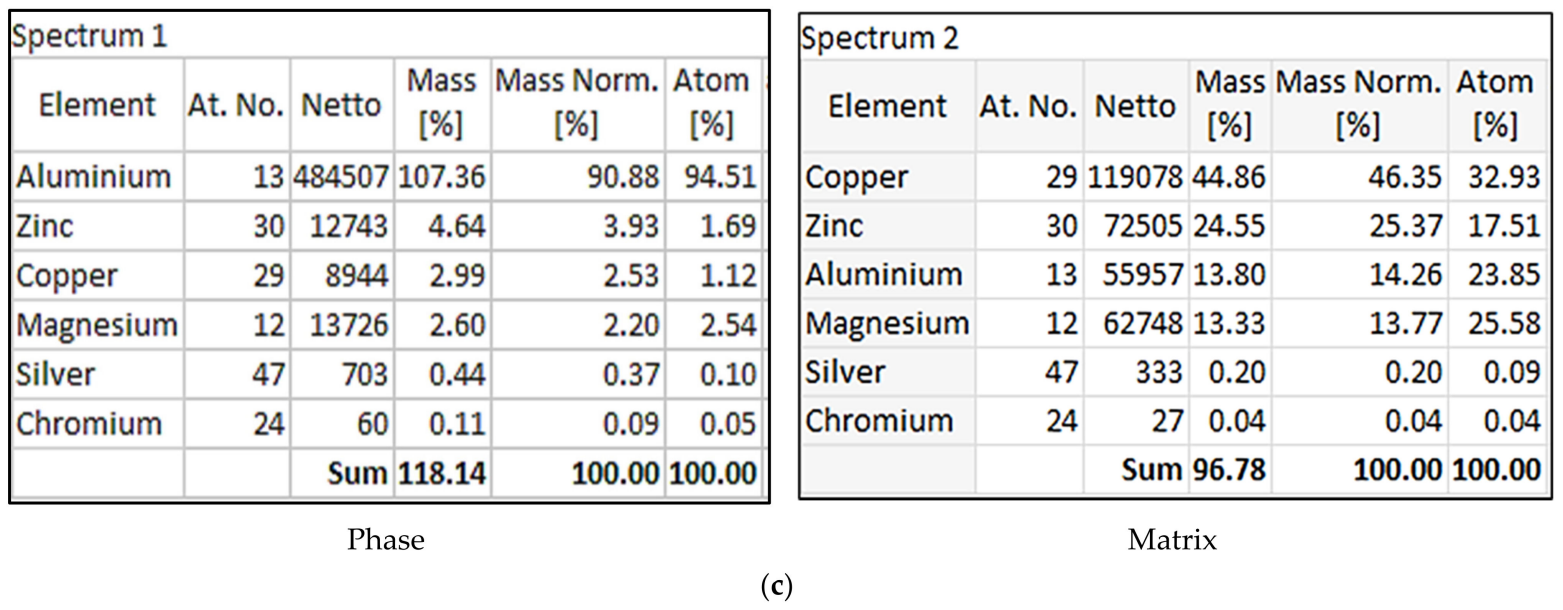
(**a**) SEM images and (**b**) elemental maps showing the distribution of Al, Mg, Cu, and Zn in the AlMgCuZn eutectic phase and (**c**) EDS results of the as-cast Al 7075-Sc alloy sample.

The 7075-aluminum alloy contains a range of alloying elements in significant quantities, and segregation takes place during the solidification process. This segregation contributes to the development of a coarse non-equilibrium eutectic phase located at the grain boundary. SEM and EDS images of the as-cast eutectic structure of Al 7075-Sc alloy are shown in Figure 8a,b. The small needle-like particles observed around the non-equilibrium eutectic phase (see the high magnification image to the right in Figure 8a), are generally considered to be the MgZn_2_ phase, separated by the precipitate free zone (PFZ) from the eutectic AlMgCuZn phase.

**Figure 9:**
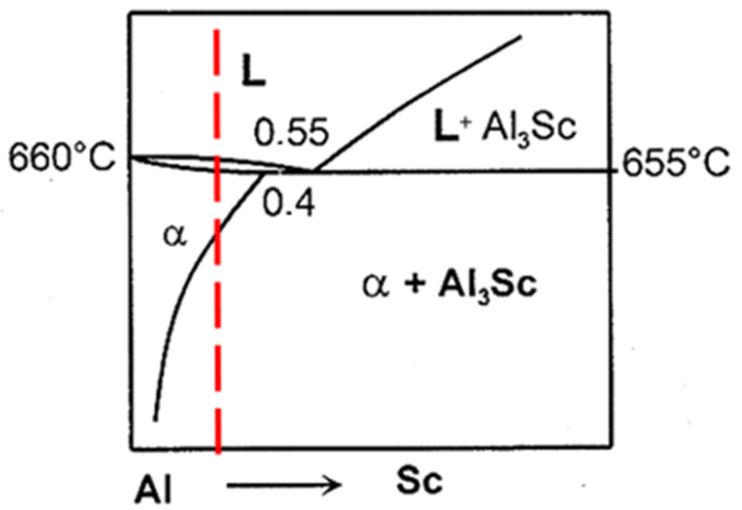
Equilibrium phase diagram for the Al–Sc system where the red dashed line corresponds to the present alloy composition.

**Figure 10:**
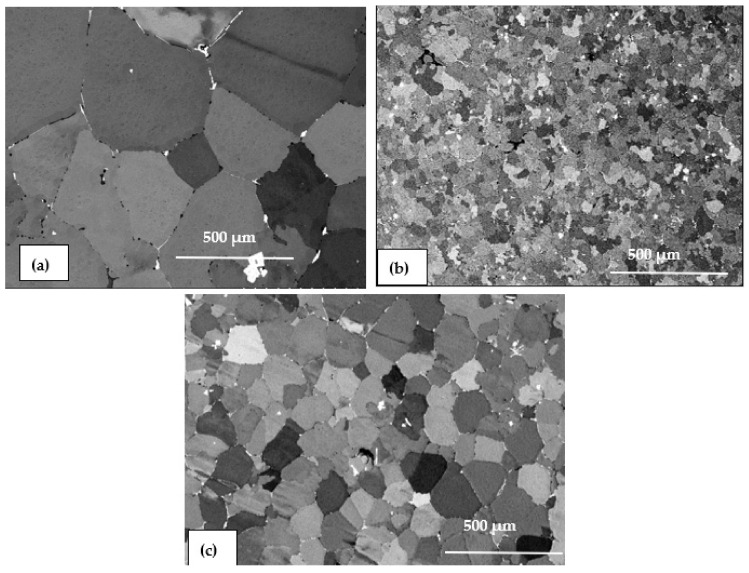
Backscattered electron images showing the structure of as-cast alloy samples: (**a**) base alloy (coded A)—overage grain size is about 500 µm and (**b**) Al–Sc alloy with 0.11% Sc (coded B) average grain size is about 50 µm, and (**c**) Al–Li–Sc alloy (coded C)—average grain size is about 150 µm.

**Figure 11:**
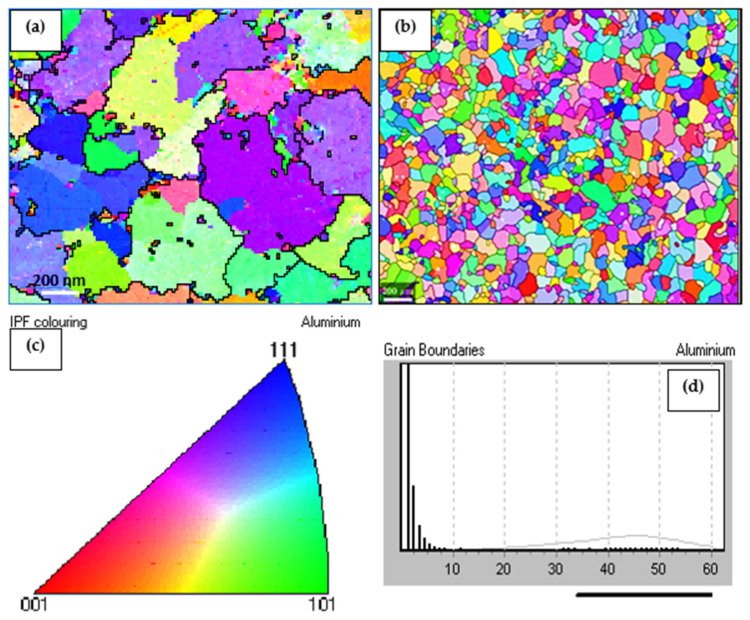
Grain size and orientation maps of as cast: (**a**) 7075 base alloy; (**b**) Al-Sc alloy; (**c**) IPF of (**c**), (**d**) grain boundaries.

Based on the Al-Sc phase diagram displayed in Figure 9, when the scandium content reaches the eutectic composition of 0.55 wt.% Sc, primary particles of L1_2_ Al_3_Sc are formed in a eutectic reaction: L + Al_3_Sc → (Al) at 665 °C, and they in turn act as nucleation sites for α-Al in the molten metal during solidification. The potent grain refining efficiency of L1_2_ Al_3_Sc for α-Al may be interpreted by the near identical crystal structures of Al_3_Sc and α-Al as well as to the very low lattice misfit between the two phases (≈1.5%) [35]. Krug et al. [36] examined the distributions of precipitates in heat-treated Al-Li-Sc and Al-Li-Sc-Yb alloys, and found that nano-sized α-Al_3_(Li,Sc,Yb)(L1_2_) precipitates were formed after isothermal aging at 325 °C, where, at times, δ′-Al_3_Li(L1_2_) shells also formed on these precipitates after further aging at 170 °C. Figure 10 displays the effectiveness of Sc as a strong grain refiner for the present alloys and confirmed by the EBSD technique (Figure 11). Due to the interaction between Li and Sc, this would reduce the amount of Sc available for grain refining, thus resulting in coarser grains in alloy C, compared to those in alloy B. The IPF map (Figure 11c) indicates the randomness of grain orientation.

**Figure 12:**
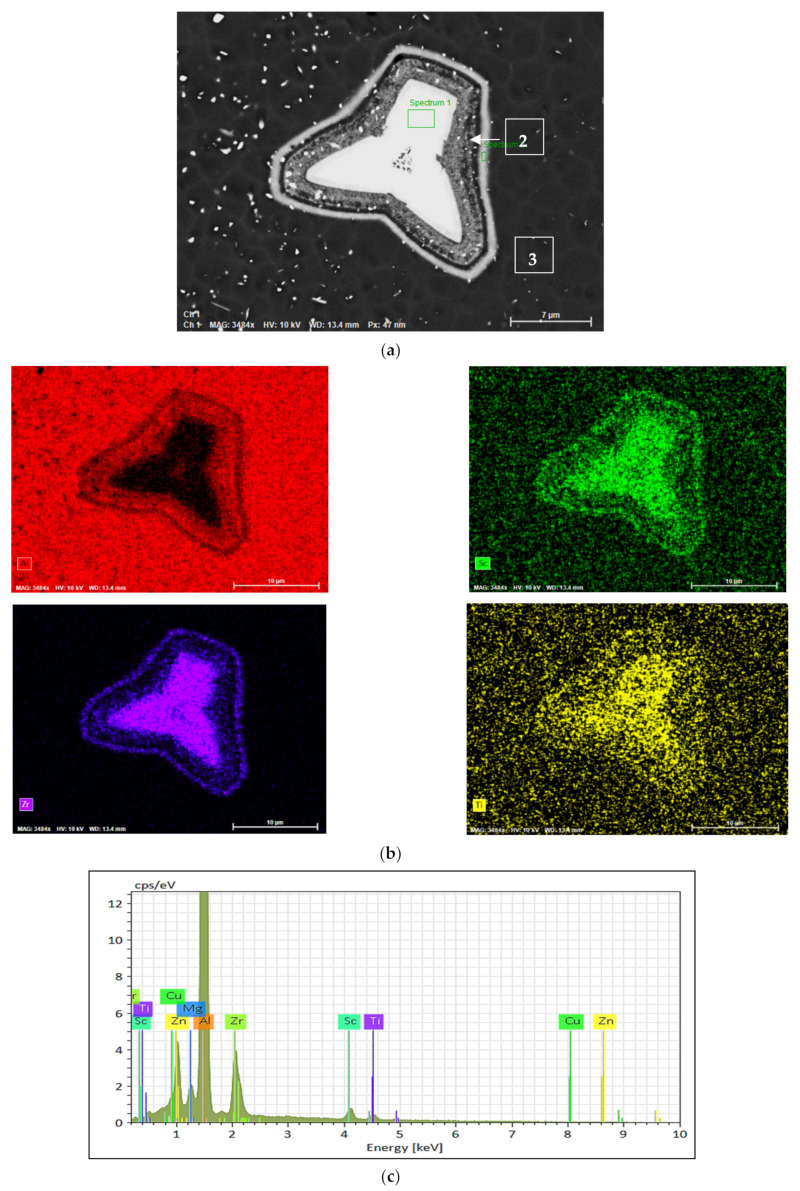

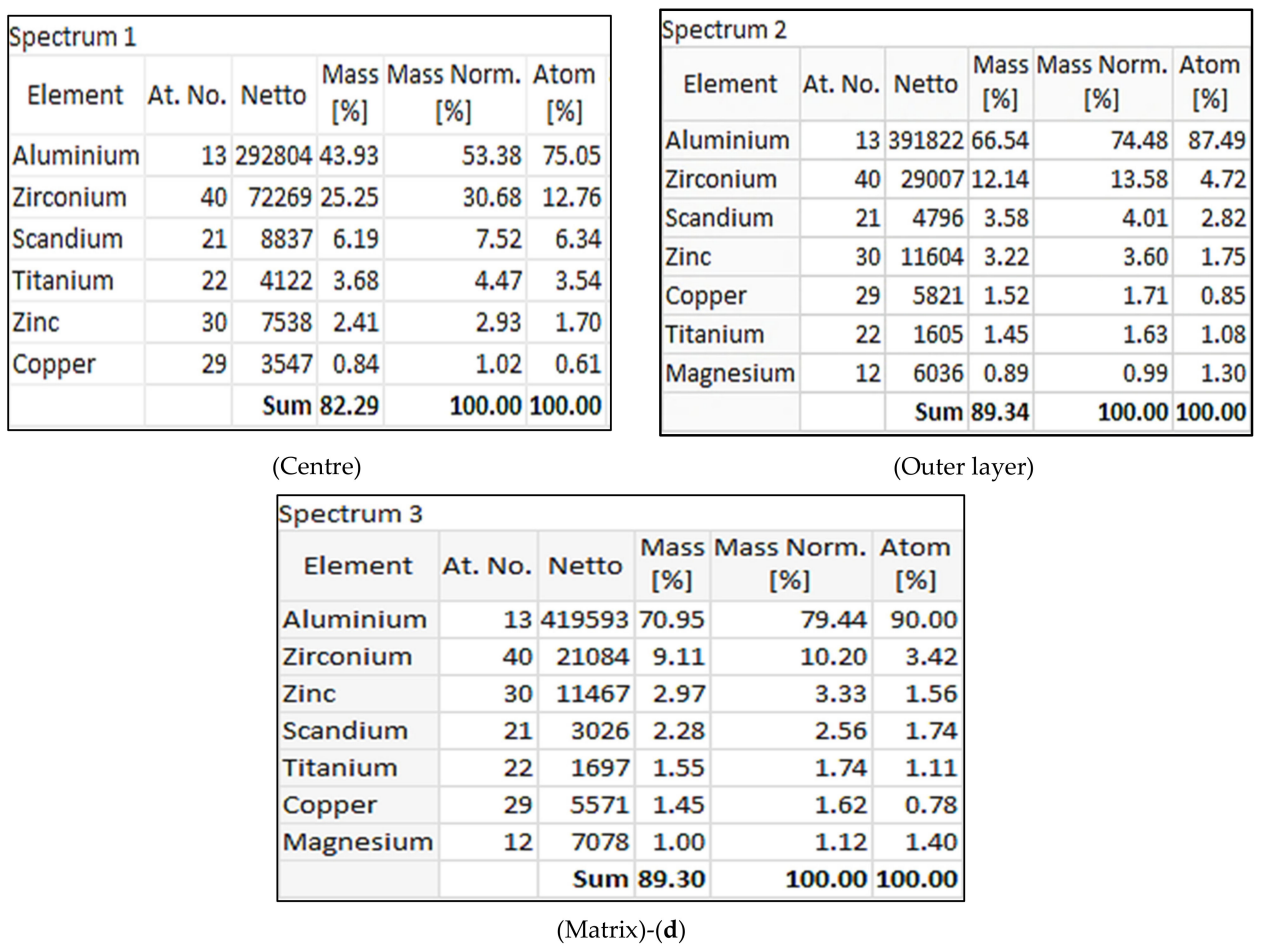
Precipitation of Al_3_(Sc, Zr, and Ti) phase particles during solidification of the Al 7075-Sc alloy at 0.15 °C/s: (**a**) backscattered electron image, (**b**) X-ray images of the distribution of Al, Sc, Zr, and Ti elements in (**a**); (**c**) EDS spectrum corresponding to the area marked 1 in (**a**); and (**d**) EDS data analysis.

Figure 12a shows the shape of the observed Al_3_(Sc, Zr, and Ti) phase particles formed during the course of solidification (0.15 °C/s) whereas Figure 11b displays the distribution of the main elements in the particle seen in Figure 12a. Figure 12c displays the EDS spectrum taken from the rectangular area marked Spectrum 1 in Figure 12a. As can be seen from Figure 12a, the particle is not solid. Rather, it is formed over several layers with different compositions, as noted from the results obtained from the spectra corresponding to points 1, 2, and 3 marked in Figure 12a and displayed collectively in Figure 12d; the maximum concentrations of Zr and Sc are found at the center of the particle. The primary phase particles of Al_3_(Sc_x_Zr_1−x_) normally appear in the form of a star-like shape, as seen in Figure 12a.

The primary dispersoid particles of Al_3_(Sc_x_Zr_1−x_) have greater refining efficiency than the primary particles of Al_3_Sc or Al_3_Zr [37]. The Al_3_Sc phase can also dissolve up to 5% of the titanium to produce Al_3_(Sc_1−x_Ti_x_) so that, in the presence of titanium combined with zirconium, Al_3_(Sc_1−x−y_Zr_x_Ti_y_) may be formed. The primary phase particles of Al_3_(Sc_x_Zr_1−x_) normally appear in two dimensions in the form of a star-like shape, as shown in Figure 12a.

**Figure 13:**
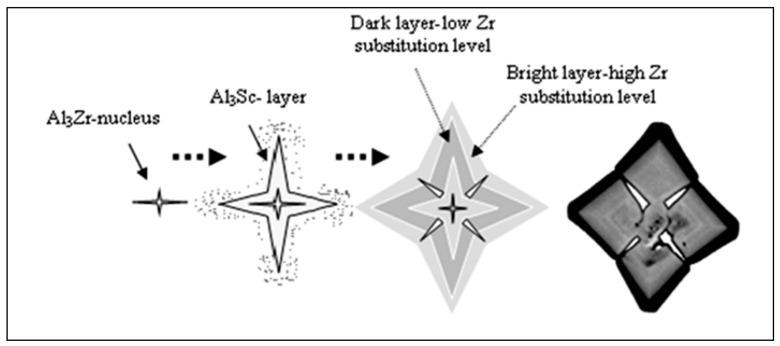
Schematic diagram showing the nucleation and growth sequence of the Al_3_(Sc_1−x_Zr_x_)-star-like phase particle.

It is believed that since the Al_3_Zr phase nucleates as the primary phase, the star phase grows predominantly through the precipitation of Al_3_Sc on the surface of the Al_3_Zr in the form of layers displaying different degrees of brightness. The variation in brightness from one layer to another is ascribed to the variation in the amount of Zr atoms replacing Sc atoms in the Al_3_Sc phase. An alternate mechanism may thus be considered more feasible than the first. A schematic sketch of the formation sequence for the star phase is provided in Figure 13 based on this second mechanism.

**Figure 14:**
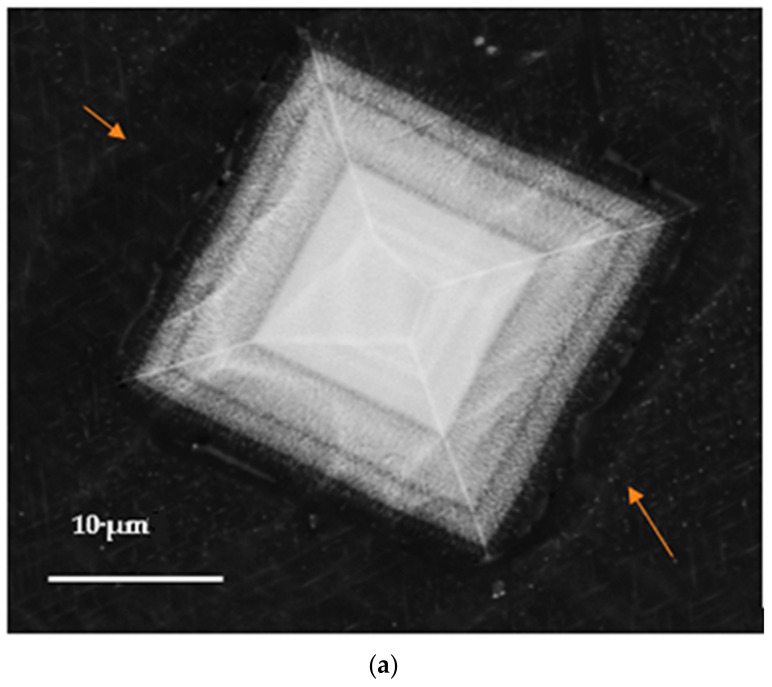

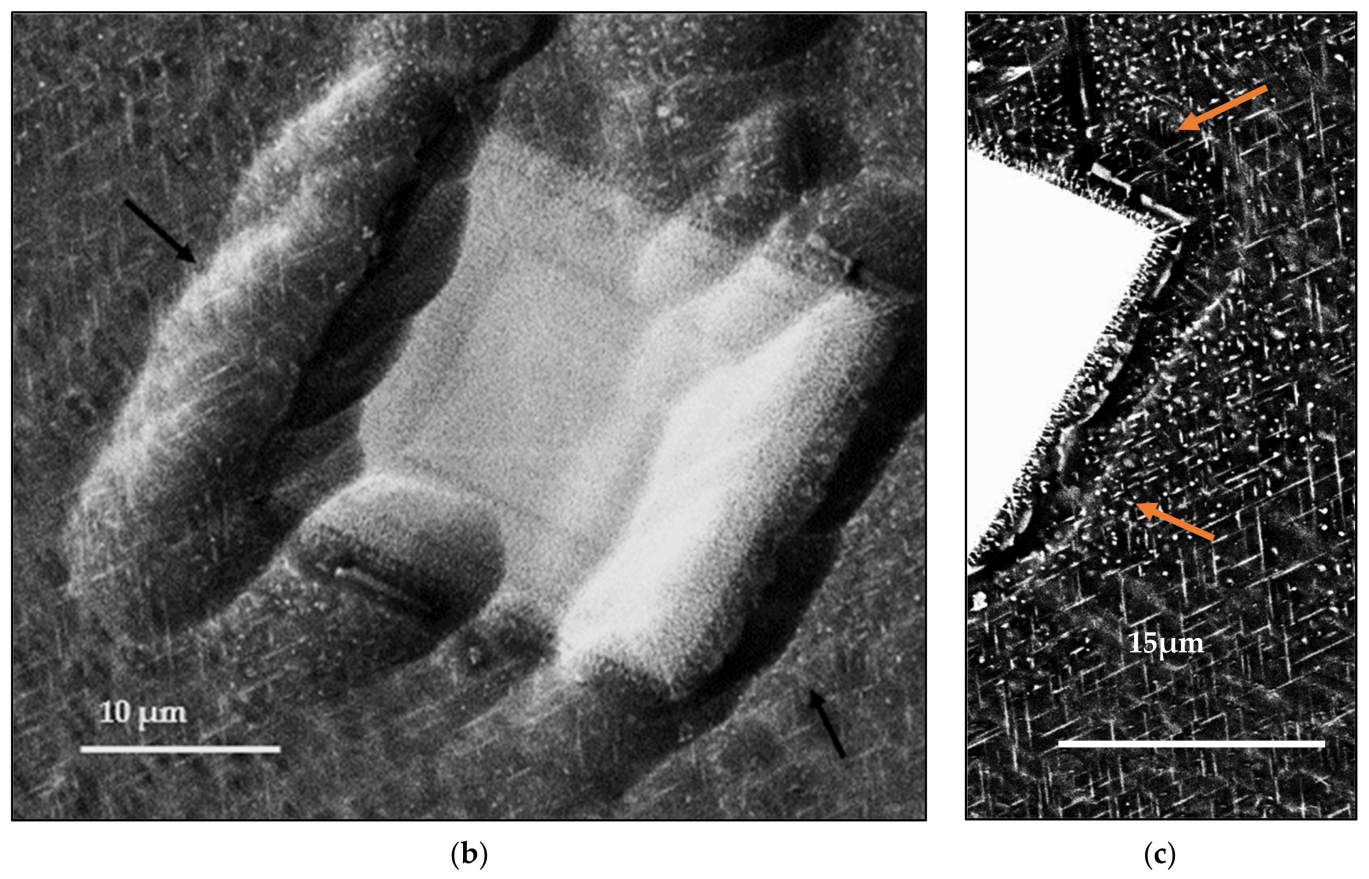
(**a**) Precipitation of AlMgCuZn phase particles on the boundaries of a pre-existing Sc-dispersoid; (**b**) the difference in level between the star-like particle and the surrounding aluminum matrix. Orange arrows in (**a**) point to grain boundaries whereas the orange arrow shows the width of the precipitate-free zone on both sides of the grain boundary; note the presence of intense precipitation around the Al_3_Sc particle. Black arrows in (**b**) point to the difference in level between both Al_3_Sc particles and the surrounding matrix; (**c**) an enlarged area of (**a**) showing the precipitate-free zone.

Figure 14a reveals the presence of an Sc-rich dispersoid in T7-treated Al 7075-Sc alloy (alloy aged at 280 °C) where fine precipitation particles are observed decorating the boundaries of a star-like precipitate whereas Figure 14b depicts the difference in topography between the particle and matrix (thick black arrow). Marquis and Seidman [38] confirmed the possibility of such observations in Al-0.1%Sc on a nanoscale when the alloy was aged at temperatures as high as 380 °C for times up to 250 h. The authors interpreted the formation of these particles as due to the splitting of coherent precipitates in an incommensurate anisotropic system. Figure 14c is an enlarged portion of Figure 14b showing the precipitation away from the primary Al_3_Sc particle.

## 8. Conclusions

Based on the data which evolved from the above investigations, the following remarks may be made:

1. Regardless of the type of aging process, i.e., single or double, the highest aging temperature is the main parameter controlling the alloy hardness/strength;
  - Aging at 120 °C for over 24 h provides maximum strength (in the range of 175–200 VHN due to precipitation of the hardening phases in the form of ultra-fine particles;
  - Aging at higher temperatures, i.e., 180 °C or 280 °C, whether it is the first aging temperature or the second one in a double aging process, leads to a dramatic decrease in the alloy strength due to coarsening of the precipitated phase particles (65–145 VHN).
2. The precipitation of η-phase particles is associated with the formation of precipitate free zones around the pre-existing phases;
3. In the T7 condition, the presence of Al and Cu in the alloy would change the composition of the η-phase. In all cases, the Zn/Mg ratios are about unity;
4. The coarsening and spheroidization of η-phase particles takes place through the Ostwald ripening mechanism where smaller particles in solution dissolve and deposit on larger particles;
5. In Sc-containing alloys, the star phase particle consists of different layers which may be discerned by the variations in brightness across the four arms of the particle;
6. The change in the brightness from layer to layer indicates that the Zr and Sc concentrations are varied within the star phase since the atomic number of Zr (40) is higher than the atomic number of Sc (21) so a Zr-rich layer will be brighter than an Sc-rich layer;
7. Ultra-fine Al_3_Li particles were observed in the Al 7075-Sc-Li alloy. However, under T7 temper conditions (treatment #3) the hardening caused by these Al_3_Li precipitates is compromised by the alloy softening, resulting in a negligible hardening effect.

## Figures and Tables

**Table 1:** Chemical composition (wt.%) of the alloys used in this study.

Alloy Code	Alloy	Si	Mg	Cr	Mn	Fe	Cu	Zn	Zr	Ti	Ag	Li	Sc
* A	Al 7075	0.21	2.15	0.19	0.035	0.16	1.45	5.62	0.3	0.027	0.23	-	-
B	Al 7075-Sc	0.16	2.2	0.14	<0.003	0.099	1.3	6.5	0.3	0.080	0.23	0.06	0.12
C	Al 7075-Li-Sc	0.11	2.8	0.16	<0.003	0.053	1.5	6.6	0.3	0.094	0.23	2.2	0.11

* Commercial alloy.

## Data Availability

Data are contained within the article.
